# Thermal properties of Zn_2_(C_8_H_4_O_4_)_2_•C_6_H_12_N_2_ metal-organic framework compound and mirror symmetry violation of dabco molecules

**DOI:** 10.1038/s41598-017-11326-6

**Published:** 2017-09-14

**Authors:** Svetlana G. Kozlova, Svyatoslav P. Gabuda

**Affiliations:** 1 Nikolaev Institute of Inorganic Chemistry, Siberian Branch of the Russian Academy of Sciences, Academician Lavrentiev Avenue 3, 630090 Novosibirsk, Russian Federation; 2 Novosibirsk State University, Pirogova Street 2, 630090 Novosibirsk, Russian Federation

## Abstract

Thermal properties of Zn_2_(C_8_H_4_O_4_)_2_•C_6_H_12_N_2_ metal-organic framework compound at 8–300 K suggest the possibility of subbarrier tunnelling transitions between left-twisted (S) and right-twisted (R) forms of C_6_H_12_N_2_ dabco molecules with D_3_ point symmetry. The data agree with those obtained for the temperature behavior of nuclear spin-lattice relaxation times. It is shown that there is a temperature range where the transitions are stopped. Therefore, Zn_2_(C_8_H_4_O_4_)_2_•C_6_H_12_N_2_ and related compounds are interesting objects to study the effect of spontaneous mirror-symmetry breaking and stabilization of chiral isomeric molecules in solids at low temperatures.

## Introduction

Chirality-related interactions are demonstrated by chiral molecules, i.e. those that can exist in both left- and right-handed forms. According to molecular quantum mechanics, such molecules appear in their ground state which is a symmetrical superposition of these two chiral states. However, biological systems are asymmetric (superselection phenomenon) due to fundamental parity violations^1–3^ or the concept of environmental decoherence^4–6^. As it was discussed earlier, it would be of interest to study the stabilization of chiral molecules in solids at low temperatures to simulate the conditions of the cold scenario^7^. However, the expected fundamental conclusions are still not supported by detailed analysis of the interactions within crystal structures where the stabilization effect is not suppressed by other impacts^8, 9^. In this respect, of high interest are metal-organic framework compounds with large pores, open internal channels, and large internal surface areas^10–12^.

**Figure 1:**
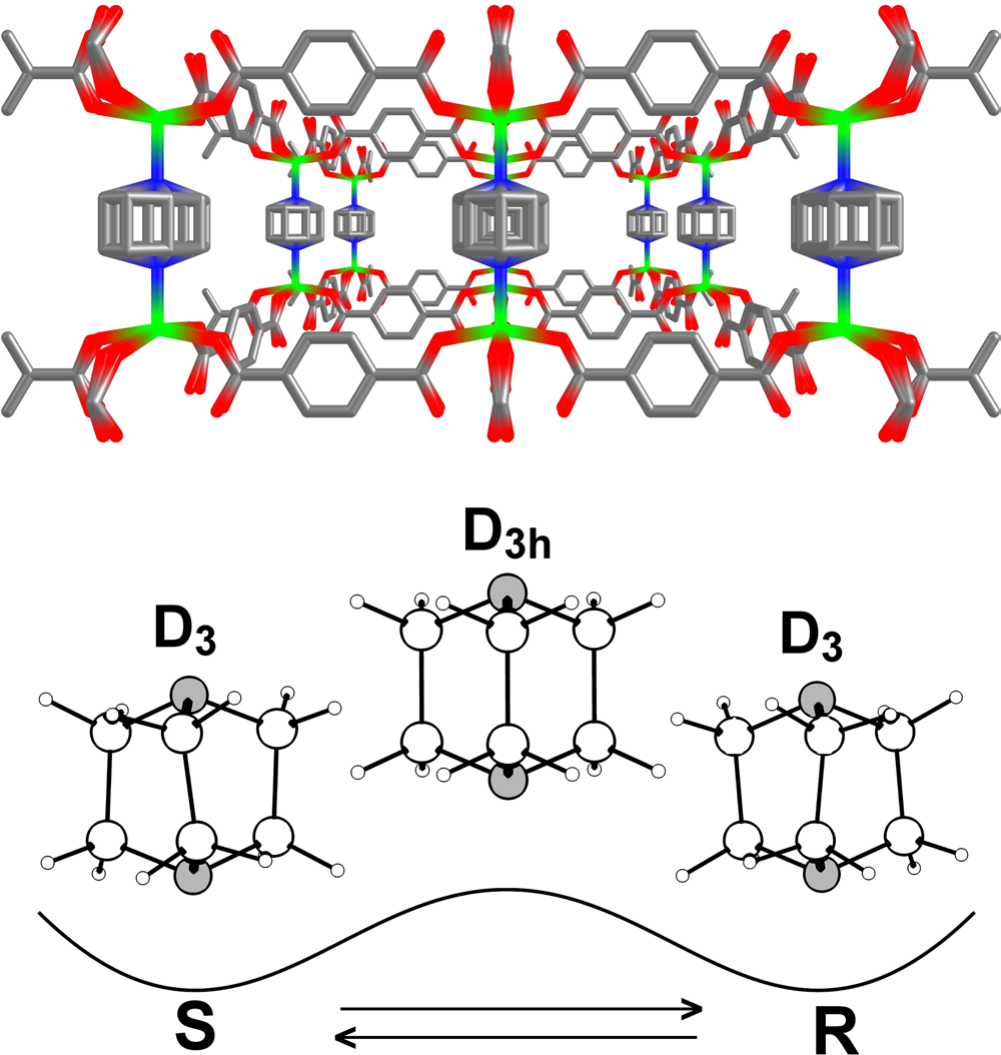
Crystal structure of Zn_2_(C_8_H_4_O_4_)_2_• C_6_H_12_N_2_, space group *P*4/*mmm*; *a* = 10.93, *c* = 9.61 Å; Z = 1; T = 223 К^11^. Pillar dabco molecules are shown as dynamically disordered (top). Left- and right-twisted D_3_ and untwisted D_3h_ conformations of dabco molecules (bottom).

One such typical example is Zn_2_(C_8_H_4_O_4_)_2_•C_6_H_12_N_2_ crystal. It is composed of tetragonal layers of zinc terephthalic acid Zn(C_8_H_4_O_4_) linked by 1,4-diazabicyclo[2.2.2] octane molecules (C_6_H_12_N_2_, or dabco). Dabco molecules have three isomeric forms: one untwisted form with D_3h_ symmetry, one left-twisted (S) and one right-twisted (R) form with D_3_ symmetry each (Fig. 1). Dabco molecules are dynamically disordered around the ***c*** axis of Zn_2_(C_8_H_4_O_4_)_2_•C_6_H_12_N crystal. The distance between them is ~ 7 Å, so they have no direct contacts with each other^11^.

According to our calculations of isolated dabco molecules, their degenerate energy states correspond to twisted D_3_(S) and D_3_(R) forms, and the energy barrier (E_a_) between these two states can vary between 0 and 0.36 kJ/mol depending on the external conditions^13^. Consequently, D_3_(S) ↔ D_3_(R) transforms can happen as: (1) barrier-free transitions; (2) activation transitions to overcome the barrier; (3) subbarrier tunnelling transitions. Low activation barrier E_a_ between D_3_(S) and D_3_(R) forms makes prospective studying tunnelling transitions between these forms, in contrast to those between L- and D-forms of amino acids in solids with E_a_ ~ 300 kJ/mol barrier^9^.

The untwisted D_3h_ state of the dabco molecule corresponds to its transition state and therefore its energy is equal to the value of the energy barrier E_a_ (Fig. 1)^13^. However, this state can be stable in crystals, and the untwisted molecule can move using both the activation and the tunnelling mechanisms.

As it was discovered earlier, there are three phase transitions in Zn_2_(C_8_H_4_O_4_)_2_•C_6_H_12_N_2_^14–16^. Temperature behavior of spin-lattice relaxation times studied with ^1^H NMR T_1_ demonstrates the effects of tunnelling transitions starting between D_3_(S) and D_3_(R) forms of dabco molecules at ~165 K, the violation of these transitions at ~60 K, and substantial difference between the values of spin-lattice relaxation data for dabco conformers at <25 K. In this work we analyze thermal energies of dabco molecules in Zn_2_(C_8_H_4_O_4_)_2_•C_6_H_12_N_2_ to show that these effects are thermally possible.

## Theoretical background

Over-barrier transitions (activation) are characterized by the correlation time

1 $${\tau }_{{\rm{a}}}={\tau }_{{\rm{a}}0}\cdot {\rm{e}}{\rm{x}}{\rm{p}}(\frac{{{\rm{E}}}_{{\rm{a}}}}{{{\rm{k}}}_{{\rm{B}}}\cdot {\rm{T}}}),$$

where k_B_ is the Boltzmann constant, τ_a0_ is the pre-exponential factor for the Arrhenius law, T is the temperature.

Subbarrier tunnelling is described by the Shrödinger equation and is characterized by the correlation time

2 $${\tau }_{{\rm{t}}}={\tau }_{{\rm{t}}0}\cdot {\rm{e}}{\rm{x}}{\rm{p}}[\frac{2{\rm{L}}}{\hslash }\sqrt{2{\rm{m}}({{\rm{E}}}_{{\rm{a}}}-E)}],$$

where m is the mass of the tunnelling particle, τ_t0_ is the pre-exponential factor for tunnelling transitions (inverse vibrational frequency of the particle in the potential well), E is the kinetic (thermal) energy of the particle, L is the width of the activation barrier, ħ is the Planck constant.

As is well known, thermal energy of atoms and molecules is determined by the temperature of the solid and can be calculated as E = C_p_﻿·T, where C_p_ is the thermal capacity of the solid at constant pressure.

In solids, thermal energy is unevenly distributed between atoms and molecules, and at each moment the amplitude and the energy of thermal vibrations for some part of particles can be higher or lower than their average values. To make the transitions possible, some thermal energy is needed. Activation transitions require that some part of particles have E > E_a_, while tunnelling transitions proceed at E < E_a_.

We assume that tunneling and activation transitions have the same reaction coordinate. Then for tunnelling transitions with reaction coordinate coinciding with that of reorientational motion (maintaining D_3h_ and D_3_ symmetries), the minimum distances between the atoms are D^H^ = 1.08 Å (for hydrogen atoms) and D^C^ = 0.65 Å (for carbon atoms). For tunnelling transitions between D_3_(S) and D_3_(R) these distances are D^H^ = 0.66 Å and D^C^ = 0.15 Å^13^. Both D^C^ values are smaller than the covalent radius of the carbon atom (r^C^ ~ 0.70 Å). Hence, we can assume that carbon atoms change their positions during tunnelling without having to overcome a barrier. D^H^ values exceed the covalent radius of the hydrogen atom (r^H^ ~ 0.30 Å). In this case, the barrier has finite width L and reaches its minimum of ~0.06 Å for tunnelling transitions between D_3_(S) and D_3_(R) dabco forms. The comparison of distances between hydrogen and carbon atoms for both types of tunnelling transitions suggests that the tunneling between twisted forms has the highest probability.

The activation barrier between these two forms in Zn_2_(C_8_H_4_O_4_)_2_•C_6_H_12_N_2_ crystal is unknown. Moreover, the width and the height of the activation barrier fluctuate due to thermal vibrations. They can change when the temperature decreases and consequently affect the tunneling processes. Nonetheless, the value of the parabolic barrier can be estimated as^7^:

3 $${{\rm{E}}}_{{\rm{a}}}=2{\rm{m}}{({{\rm{T}}}_{{\rm{c}}}\cdot {{\rm{k}}}_{{\rm{B}}}\cdot {\rm{L}}\cdot \pi /\hslash )}^{2}=7.7\,\,{\rm{k}}{\rm{J}}/{\rm{m}}{\rm{o}}{\rm{l}},$$

where T_c_ is the onset temperature of the tunnelling processes (in our case, 165 K)^14–16^, L = 0.06 Å, and m is the particle mass. In our case, *m* is the sum of masses of 12 hydrogen atoms. Carbon atoms can be excluded because of their barrier-free motion, and nitrogen atoms are not involved in tunnelling transitions because they are located along C_3_ axis of the dabco molecule.

## Results and Discussion

**Figure 2:**
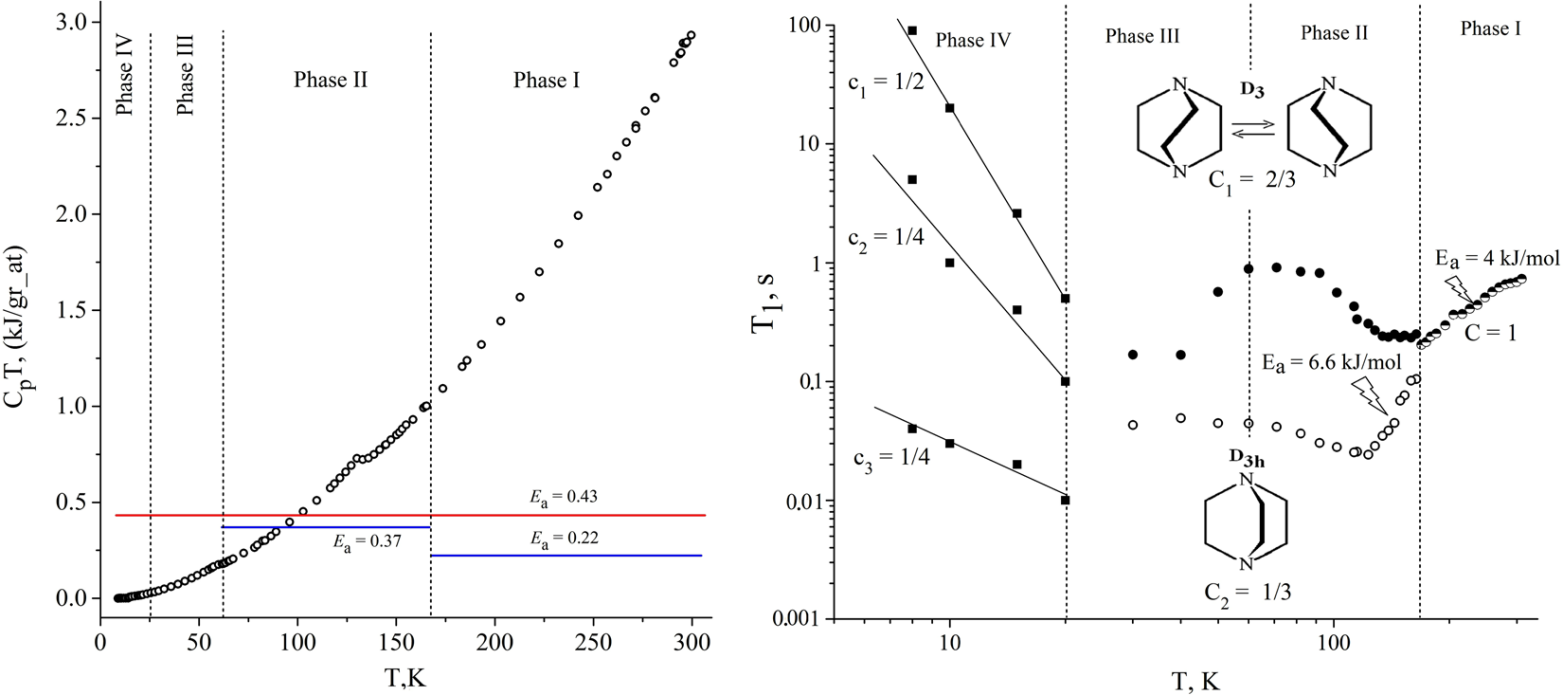
Left: temperature dependence of the thermal energy C_p_·﻿T in Zn_2_(C_8_H_4_O_4_)_2_•﻿C_6_H_12_N_2_ (о). Red horizontal line corresponds to the activation barrier (*E*_a_) between energy minima of D_3_(S) and D_3_(R) forms of dabco molecules. Blue horizontal lines correspond to the experimental activation barriers (*E*_a_) obtained for the reorientation of dabco molecules in Phases I and II. Right: temperature dependence of ^1^H NMR T_1_ in Zn_2_(C_8_H_4_O_4_)_2_•﻿C_6_H_12_N_2_ presented in the double logarithmic scale. C, C_i_ and c_i_ (i = 1, 2, 3) are the fractions of the components in the FID. E_a_ are experimental activation barriers for the reorientation of dabco molecules. Vertical dashed arrows indicate the position of phase transition temperatures according to NMR data^14–16^.

Figure 2 presents temperature dependence C_p_·﻿T, where C_p_ is the thermal capacity of Zn_2_(C_8_H_4_O_4_)_2_•C_6_H_12_N_2_ obtained in ref. 17. The same figure shows *E*_a_ values for dabco molecules. Values *E*_a_ = 0.22 kJ/gr_at and *E*_a_ = 0.37 kJ/gr_at correspond to experimental values E_a_ = 4.0 kJ/mol and E_a_ = 6.6 kJ/mol, and *E*_a_ = 0.43 kJ/gr_at corresponds to the calculated E_a_ = 7.7 kJ/mol, respectively. *E*_a_ values are assumed to correspond to activation energies only of hydrogen and carbon atoms of the dabco molecule, and nitrogen atoms are not involved in the reorientation. All values of C_p_·﻿T and E_a_ were normalized to one gram-atom to make easier comparison of the results obtained from different methods.

Since in Phase I thermal energy C_p_·﻿T > 0.43 > 0.22 kJ/gr_at, above T_c_ all dabco conformers can overcome barriers *E*_a_ = 0.43 kJ/gr_at and *E*_a_ = 0.22 kJ/gr_at through the activation mechanism, which agrees with T_1_ NMR data (Fig. 2). The temperature dependence of ^1^Н NMR T_1_ in Zn_2_(C_8_H_4_O_4_)_2_•C_6_H_12_N_2_ at 310–165 K obeys the classical theory of nuclear spin-lattice relaxation and is characterized by a single-exponential recovery of the free induction decay (FID)^18, 19^. The activation mobility of dabco molecules is characterized by one barrier E_a_ = 4 kJ/mol and τ_a0_ = 1.1·﻿10^−14^ s. It means that in Phase I either two twisted forms and one untwisted form of dabco molecules are indistinguishable in their energies, or that the system has only one of three conformations (e.g., untwisted) as energetically excited^14, 16^.

Thermal energy C_p_·﻿T varies in Phase II from 1.0 to 0.2 kJ/gr_at. These thermal energies make it impossible for all particles to overcome the barrier with *E*_a_ = 0.43 kJ/gr_at by the activation mechanism, as well as the barrier *E*_a_ = 0.37 kJ/gr_at. Hence, this phase suggests tunnelling transitions. Indeed, at 165–60 K the activation mobility of particles is violated. Firstly, the double-exponential recovery of FID is observed^14, 16^. The contribution of FIDs characterized by longer time T_1_^L^ and shorter time T_1_^S^ to the total magnetization are С_1_ = 2/3 and С_2_ = 1/3, respectively. This double-exponential recovery of FID may indicate that activation mobility of dabco molecules is hindered and that energy states of their conformers become different. From the obtained С_1_ and С_2_ values we can conclude that the energy states were equally stabilized for each conformer. Since D_3_(S) and D_3_(R) forms are characterized by the same time T_1_^L^, they are indistinguishable in energy. These dabco molecules can make tunnelling transitions under the barrier E_a_ = 7.7 kJ/mol. Their states are energetically more favorable than those of the untwisted form. This follows from the fact that if molecules are distributed over their energy states in crystals according to the Boltzmann statistics, then the energy population for the untwisted form (C_2_) in Zn_2_(C_8_H_4_O_4_)_2_•C_6_H_12_N_2_ turns out to be lower than that of two twisted forms (C_1_). The conclusion correlates with quantum chemical calculations which show that the untwisted form of a free dabco molecule (D_3h_ symmetry) is its transition state^13^. Secondly, in the range 165–120 K T_1_^L^ is virtually temperature independent, which indicates tunnelling transitions^14, 16^. The T_1_^S^ behavior at these temperatures corresponds to the activation process with E_a_ = 6.6 kJ/mol and τ_a0_ = 0.3·﻿10^−14^ s. Therefore, untwisted molecules continue to participate in activation transitions. Below 100 K dabco molecules are capable mostly of tunnelling transitions, since C_p_·﻿T < 0.37 kJ/gr_at. Therefore, the structure of untwisted dabco molecules cannot correspond to the stable state and must get its symmetry lower.

As follows from Fig. 2, in Phase III tunnelling transitions are possible. On the other hand, due to possible asymmetry of the double-well potential for D_3_(S) and D_3_(R) forms the transitions can be stopped. This is indicated by the behavior of anomalous part of the heat capacity of Zn_2_(C_8_H_4_O_4_)_2_•C_6_H_12_N_2_ at the second order phase transitions ~60 K^20^. Below ~60 K, the anomalous part of the specific heat demonstrates exponential behavior, which suggests tunnelling of less stable right-enantiomers into more stable left-enantiomers (Salam model)^2, 3, 20^.

In Phase IV, thermal energy is C_p_·﻿T ≤ 0.02 kJ/gr_at. In this case, only tunnelling transitions are possible, and, according to equation (2), time τ_t_ must reach its highest value here. However, the behavior of T_1_ (Fig. 2) does not correspond to classical views on tunnelling processes^7, 21, 22^. T_1_ grows when the temperature decreases (like T_1_ during activation transitions). But there is no consistent activation mobility of the particles neither. Experimental data on time dependences of FID for these temperatures makes it possible to distinguish at least three components (c_1_, c_2_, c_3_) characterized by three different T_1_ values (Fig. 2). This can mean that D_3_(S) and D_3_(R) energies are not equal at the lowest temperatures and that the system as a whole must be characterized by chiral polarization^14–16^. The discovered difference between Т_1_ times can be considered as an analogue of the previously discovered effect when the multiplicity of the NMR spectrum is doubled when passing from a optically inactive (racemic) to optically active mixtures of chiral isomers^23, 24^.

As was shown earlier, the mechanism of phase transition from Phase III to Phase IV in our model can be associated with the ordered packing of untunnelling and non-reorienting D_3_(S) and D_3_(R) dabco molecules^14–16^. In this case, violation of D_3_(S) ↔ D_3_(R) symmetry can be due to random factors similar to those affecting the precipitation of R- and S-forms of optically active crystals from racemates^25^. However, the fact that there are three values c_1_, c_2_, and c_3_ (Fig. 2) obtained from the analysis of FID suggests some ambiguity of the proposed model. We can assume that further temperature decrease should lead to further phase transitions and molecular ordering. Also, some additional mechanism to cause non-exponential FID and nuclear spin-lattice relaxations is also possible^16^.

Note that we do not consider here the mobility of C_8_H_4_O_4_^2−^ anions, because their high activation barrier (>36 kJ/mol) makes them perform only slow reorientations about the second-order axis. There are no fluctuations of intramolecular dipole-dipole interactions, the fluctuations of intermolecular dipole-dipole interactions are small, and reorientation of C_8_H_4_O_4_^2−^ anions is not evidenced by ^1^Н NMR T_1_ measurements^14, 26–28^.

## Conclusions

Thermal properties of Zn_2_(C_8_H_4_O_4_)_2_•C_6_H_12_N_2_ crystals suggest that there is a possibility that mirror symmetry can be violated between D_3_(S) and D_3_(R) forms of dabco molecules. Most interesting are the lowest temperatures where all conformers can be stabilized in their local positions.

Structural transformations associated with the ordering of dynamically disordered dabco molecules in Zn_2_(C_8_H_4_O_4_)_2_•C_6_H_12_N_2_ during phase transitions can, in principle, be characterized using the approaches described in refs 29 and 30. Here we can only describe the structures expected in different phases. In the high-temperature Phase I, twisted and untwisted dabco molecules are fully disordered. In the Phase II, the crystal structure is built of the chains of dabco molecules, some of which are composed only of untwisted forms and other only of twisted forms. In Phase III, the twisting of dabco enantiomers is expected to be hindered. Finally, when the interaction between the chains becomes prevailing, the crystal structure is supposed to be chirally ordered in Phase IV.

Note that according to a recent study of [Zn_2_(C_8_H_4_O_4_)_2_•C_6_H_12_N_2_] properties, the Phase II → Phase I transition can be interpreted as an order-disorder phase transition associated with some structural disorder of C_8_H_4_O_4_^2−^ anions^12^. However, dabco molecules remain dynamically disordered and their role in the phase transition is not defined.

Thus, in our opinion, metal-organic framework compound [Zn_2_(C_8_H_4_O_4_)_2_•C_6_H_12_N_2_] and related crystals^10–12, 31^ containing racemic mixtures of chiral molecules are convenient systems to develop the approaches aimed at controlling molecular transitions from racemic to chirally polarized states. A special feature of these systems is the absence of direct contacts between chiral molecules in crystals. Such systems are interesting in terms of studying the stabilization of chiral molecules in solids at low temperatures and can serve as models for the conditions of the cold scenario of life origin on the Earth^7^.

## Methods

Heat capacity was measured at 8.98–299.57 K using a computerized vacuum adiabatic calorimeter well tested by measurements of various compounds including sorbents metal-organic framework compound [Zn_2_(C_8_H_4_O_4_)_2_•C_6_H_12_N_2_]. The details of the synthesis, experimental conditions, and heat capacity values can be found in work^17^. The ^1^H NMR spin-lattice relaxation time T_1_ of [Zn_2_(C_8_H_4_O_4_)_2_•C_6_H_12_N_2_] was measured with a Bruker SXP 4-100 device at 8–300 K and was previously analyzed in a number of works^14–16^.
